# The bipartite mitochondrial genome of *Ruizia karukerae* (Rhigonematomorpha, Nematoda)

**DOI:** 10.1038/s41598-018-25759-0

**Published:** 2018-05-10

**Authors:** Taeho Kim, Elizabeth Kern, Chungoo Park, Steven A. Nadler, Yeon Jae Bae, Joong-Ki Park

**Affiliations:** 1 Division of Environmental Science and Ecological Engineering, College of Life Sciences and Biotechnology, Korea University, Seoul, 02841 Republic of Korea; 2 Division of EcoScience, Ewha Womans University, Seoul, 03760 Republic of Korea; 3 School of Biological Sciences and Technology, Chonnam National University, Gwangju, 61186 Republic of Korea; 4 Department of Entomology and Nematology, University of California, Davis, CA 95616 USA

## Abstract

Mitochondrial genes and whole mitochondrial genome sequences are widely used as molecular markers in studying population genetics and resolving both deep and shallow nodes in phylogenetics. In animals the mitochondrial genome is generally composed of a single chromosome, but mystifying exceptions sometimes occur. We determined the complete mitochondrial genome of the millipede-parasitic nematode *Ruizia karukerae* and found its mitochondrial genome consists of two circular chromosomes, which is highly unusual in bilateral animals. Chromosome I is 7,659 bp and includes six protein-coding genes, two rRNA genes and nine tRNA genes. Chromosome II comprises 7,647 bp, with seven protein-coding genes and 16 tRNA genes. Interestingly, both chromosomes share a 1,010 bp sequence containing duplicate copies of *cox2* and three tRNA genes (*trnD*, *trnG* and *trnH*), and the nucleotide sequences between the duplicated homologous gene copies are nearly identical, suggesting a possible recent genesis for this bipartite mitochondrial genome. Given that little is known about the formation, maintenance or evolution of abnormal mitochondrial genome structures, *R. karukerae* mtDNA may provide an important early glimpse into this process.

## Introduction

The majority of metazoan mitochondrial genomes have a well-conserved structure and consist of a single circular chromosome, ranging from 14 to 20 kb and containing 37 genes: 13 protein-coding genes (PCGs) (*atp6*, *atp8*, *cob*, *cox1–3*, *nad1–6* and *nad4l*), two ribosomal RNA (rRNA) genes (*rrnL* and *rrnS*) and 22 transfer RNA (tRNA) genes^1,2^. In nematodes, mitochondrial genomes are also fairly conserved in structure and gene content, although they differ from other metazoans in some features. For example, most nematode species lack an *atp8* gene (except *Trichinella* spp. and *Trichuris* spp.^3–8^), and their tRNAs have unique secondary structures (no DHU arm in 20 tRNAs and no TΨC arm in two tRNAs, *trnS1* and *trnS2*). Complete mitochondrial genomes have been reported from more than 176 nematode species since *Caenorhabditis elegans* and *Ascaris suum* were first published in 1992^9^. Interestingly, in four nematode species the mitochondrial genome has been found to be divided into multiple chromosomes^10–13^. The reasons underlying these structural abnormalities are unclear, and the sequencing of additional mitogenomes is needed in order to better understand common features of this unusual phenomenon.

The mitochondrial genome has been used in many phylogenetic studies as a powerful molecular marker for resolving both deep and shallow nodes in various groups, including nematodes^14–17^. In recent decades, nematode mitochondrial genomes have provided independent confirmation of some phylogenetic hypotheses based on nuclear genes, and yielded insights into various evolutionary patterns such as convergent morphological evolution, and independent origins of plant parasitism^18,19^. In this study we report the complete mitochondrial genome sequence of *Ruizia karukerae*, a member of the infraorder Rhigonematomorpha, a group of about 150 named nematode species that have a direct parasitic life cycle and use millipedes as their final host^20^. The mitochondrial genome of *R. karukerae* is made up of two circular chromosomes of similar size, each encoding mostly different genes.

## Results

### The two circular mitochondrial chromosomes of *R. karukerae*

**Figure 1:**
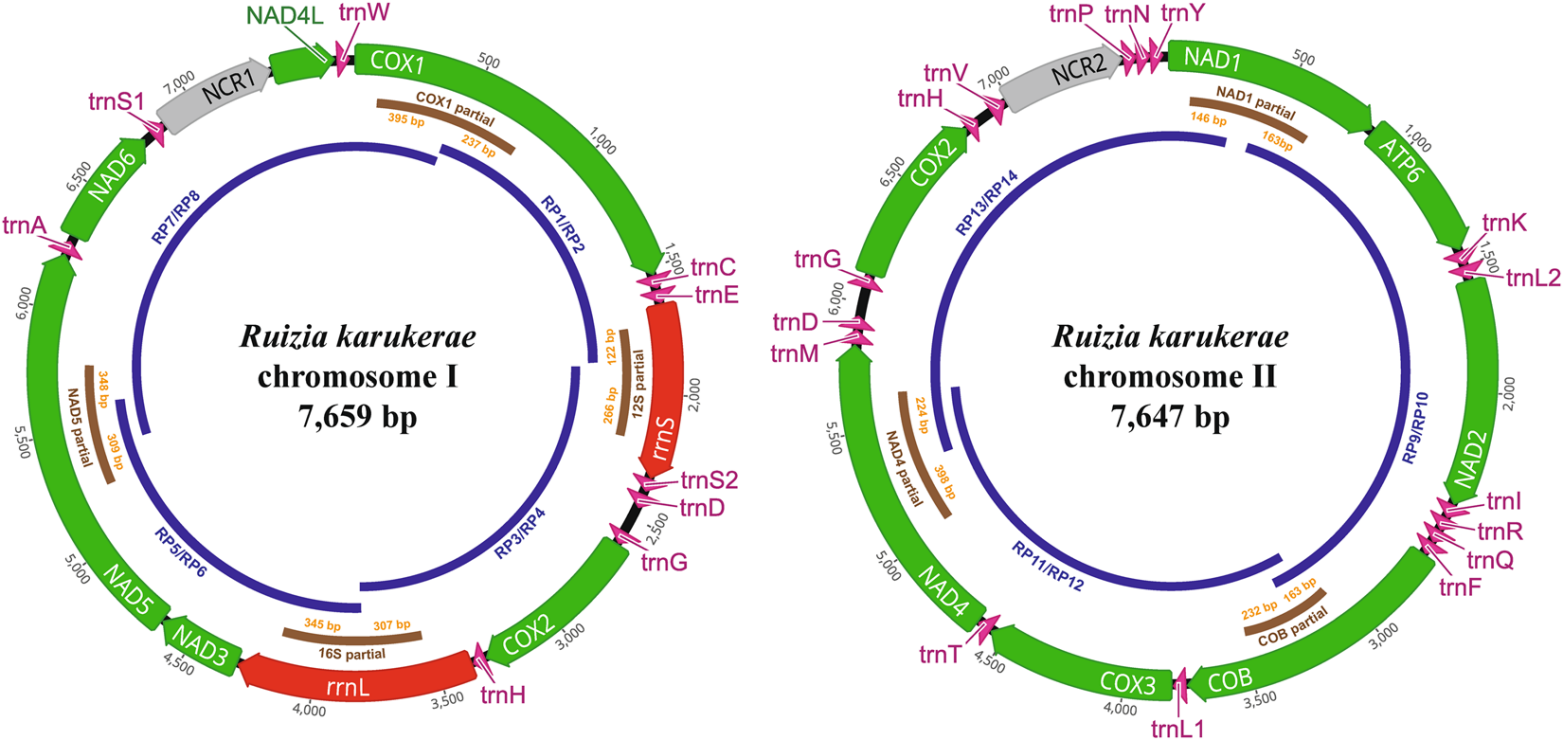
A representation of the two circular mitochondrial chromosomes of *Ruizia karukerae*. All genes are encoded in the same direction, and the 22 tRNA genes are indicated by a single-letter abbreviation. The leucine and serine tRNA genes are marked according to their anticodon sequence as L1 (*trnL1*-uag), L2 (*trnL2*-uaa), S1 (*trnS1*-ucu) and S2 (*trnS2*-uga). Primer names (see Table 2 for details) are in blue lettering, and blue curved bars indicate long PCR fragments. Brown curved bars indicate partial gene fragments obtained by 7 independent PCR reactions. Orange numbers indicate length of sequence overlap between the partial sequences and ends of long PCR fragments.

Initially, the PCR, sequencing, and assembly of four long PCR fragments [*cox1*-*rrnS* (1.5 kb), *rrnS*-*rrnL* (1.8 kb), *rrnL*-*nad5* (1.7 kb) and *nad5*-*cox1* (2.7 kb)] and partial fragment sequences (*cox1*, *rrnS*, *rrnL* and *nad5*) produced an unexpectedly small, circular molecule of mtDNA (termed chromosome I) consisting of 7,659 nucleotides and containing only six PCGs, two ribosomal genes and nine tRNA genes (Fig. 1, Table 1). Repeated attempts produced the same results. This suggested the presence of multiple chromosomes, since several key mitochondrial genes (including *cob*, *nad1* and *nad4*) were missing from the sequence of chromosome I. To locate the remaining genes not found on our assembly of chromosome I, we determined the sequences of partial fragments of *cob*, *nad1* and *nad4* individually and designed three species-specific primer sets (RP9/RP10, RP11/RP12 and RP13/RP14) (Table 2) for long PCR. These were used to amplify and sequence three overlapping fragments [*nad1*-*cob* (2.8 kb), *cob*-*nad4* (2.3 kb) and *nad4*-*nad1* (2.5 kb)], which ultimately formed a second circular mtDNA molecule (termed chromosome II), which consisted of 7,647 nucleotides and contained seven PCGs and 16 tRNA genes (Fig. 1, Table 1). The entire circular mtDNA sequences of chromosomes I and II were assembled by confirming the sequence identity in the overlapping regions (3,655 bp of overlap including 7 independently amplified fragments with overlaps ranging from 122~398 bp) between the long PCR fragments and the partial gene fragments (see Fig. 1 and Table 2 for details). Confirmation of the sequence identity in the overlapping regions, and the primer walking strategy used for the long PCR fragments employed in this study, support the presence of a bipartite circular mitochondrial genome (chromosomes I and II), rather than an artefact resulting from nuclear copies of mitochondrial DNA (“numts”). The sequences from chromosome I and chromosome II were deposited in GenBank (accession numbers MF509850 and MF509851, respectively).

**Table 1:** Mitochondrial genome organization of *Ruizia karukerae*.

Gene	Positions of nucleotide sequences	No. of nt (bp)	Initiation/termination codons	Intergenic sequence
**Chromosome I**				
*cox1*	1–1536	1536	ATT/TAA	−2
*trnC*	1535–1590	56		2
*trnE*	1593–1648	56		0
*rrnS*	1649–2338	690		0
*trnS2*	2339–2393	55		12
*trnD*	2406–2461	56		106
*trnG*	2568–2623	56		−1
*cox2*	2623−3312	690	ATA/TAA	−1
*trnH*	3312–3365	54		0
*rrnL*	3366–4306	941		0
*nad3*	4307–4642	336	ATT/TAA	−1
*nad5*	4642–6223	1582	ATA/T	0
*trnA*	6224–6278	55		19
*nad6*	6298–6771	474	ATA/TAA	29
*trnS1*	6801–6865	65		0
NCR	6866–7336	471		0
*nad4l*	7337–7588	252	ATT/TAA	5
*trnW*	7594–7646	53		13
**Chromosome II**				
*nad1*	1–861	861	ATT/TAA	2
*atp6*	864–1442	579	ATG/TAG	1
*trnK*	1444–1504	61		0
*trnL2*	1505–1559	55		1
*nad2*	1561–2449	889	ATA/T	0
*trnI*	2450–2509	60		0
*trnR*	2510–2563	54		−1
*trnQ*	2563–2617	55		−1
*trnF*	2617–2670	54		1
*cob*	2672–3760	1089	ATG/TAG	0
*trnL1*	3761–3815	55		−3
*cox3*	3813–4560	748	ATA/T	0
*trnT*	4561–4617	57		0
*nad4*	4618–5844	1227	ATA/TAA	−2
*trnM*	5843–5901	59		−1
*trnD*	5901–5956	56		106
*trnG*	6063–6118	56		−1
*cox2*	6118–6807	690	ATA/TAA	−1
*trnH*	6807–6860	54		57
*trnV*	6918–6987	70		0
NCR	6988–7476	489		0
*trnP*	7477–7529	53		−1
*trnN*	7529–7583	55		−2
*trnY*	7582–7635	54		12

**Table 2:** Primers used to sequence the complete mitochondrial genomes of *Ruizia karukerae*.

Primers	Sequence (5′-3′)	Source	Size of PCR fragment	Size of overlapping region (with long PCR fragment)
**Chromosome I**				
LCO1490	GGTCAACAAATCATAAAGATATTGG	^48^	655 bp	395 bp (RP7/RP8)
HCO2198	TAAACTTCAGGGTGACCAAAAAATCA			237 bp (RP1/RP2)
Nema_12S_F	GTTCCAGAATAATCGGCTA	This study	465 bp	122 bp (RP1/RP2)
Nema_12S_R	GCKATTGARGGATGYTTTGTACC			266 bp (RP3/RP4)
Nema_16S_F_2	TTAGTGTTGAAAAATCGTTC	This study	678 bp	307 bp (RP3/RP4)
Nema_16S_R	TCTYMCRAYGAAYTAAACTAATATC			345 bp (RP5/RP6)
Nema_ND5_F	GTTCATAGAAGTACTTTGGTKACTGCTG	This study	485 bp	309 bp (RP5/RP6)
Nema_ND5_R	AAGACGMWAACWATAAMHAAAAGT			348 bp (RP7/RP8)
RP1	AGTCTGCATATGGCAGGTGTTAGC	This study	1.5 kb	
RP2	GGCTACCCGGGTACTAATCCG			
RP3	CAAACTGAAGTAAATTGGCAGGTGC	This study	1.8 kb	
RP4	CAATGGATTATGCTACTTTAATGTCC			
RP5	GGACATTAAAGTAGCATAATCCATTG	This study	1.7 kb	
RP6	GATTAAATAAGGTAACTTCCCTAAACCAC			
RP7	GATAGAGGAGGATATGAAGAAGGTAGTG	This study	2.7 kb	
RP8	GAGCTAACACCTGCCATATGCAGAC			
**Chromosome II**				
Chroma_ND1_F_4	GGCTTTTGTAACTCTTTATGAGCG	This study	511 bp	146 bp (RP13/RP14)
Chroma_ND1_R_2	CCDCTNACYARYTCDCTYTC			163 bp (RP9/RP10)
Chroma_Cob_F_2	CARATRWSNTWTTGRGC	This study	358 bp	163 bp (RP9/RP10)
Chroma_Cob_R_2	TAYCAYTCNGGNACAAYATG			232 bp (RP11/RP12)
Chroma_ND4_F_1	CATGTHGARGCDCCNAC	This study	398 bp	398 bp (RP11/RP12)
Chroma_ND4_R_3	GTCCCAGCGTTAGTTAAAAATGTCA			224 bp (RP13/RP14)
RP9	GTAGAAGCCCCGACTACTGCTAG	This study	2.8 kb	
RP10	ACAAGCTTCTCCTTCCAGTCTCATG			
RP11	TGTTACATTTCTTGTTACCTTGGGC	This study	2.3 kb	
RP12	GTCCCAGCGTTAGTTAAAAATGTCA			
RP13	GTCTGTTCCAGAGGGATGGTAAAGCTCTAGC	This study	2.8 kb	
RP14	ATAACCACAAAGGCTACTGCGGGAG			

IUPAC nucleotide ambiguity codes used are W (A or T), R (A or G), K (G or T), S (C or G), Y (C or T), M (A or C), D (A, C or T), H (A, G or T) and N (A, C, G or T).

### Gene content of *R. karukerae* mtDNA

The mitochondrial genome of *R. karukerae* contains 12 PCGs (*atp6*, *cob*, *cox1-3*, *nad 1-6* and *nad4L*), two rRNA genes and 22 tRNA genes, and lacks an *atp8* gene, a common feature in nematode mitochondrial genomes with the exception of *Trichinella* spp. and *Trichuris* spp.^3–8^. In the mitochondrial genome of *R. karukerae*, chromosome I contains six PCGs, two rRNA genes and nine tRNA genes, and chromosome II includes seven PCGs and 16 tRNA genes (Fig. 1, Table 1). All genes are encoded in the same direction, as is common in other known chromadorean nematode mitochondrial genomes (except for *nad2* in *Plectus acuminatus* and *P. aquatilis*^21^). Interestingly, *cox2, trnD, trnG* and *trnH* were identified on both chromosomes (Fig. 1, Table 1). The *trnG* sequences on chromosomes I and II are identical, while the sequences of *cox2, trnD*, and *trnH* differ by one nucleotide between chromosomes. The nucleotide substitution between the two copies of the *cox2* gene is a nonsynonymous mutation: a TAT codon encoding tyrosine in chromosome I is substituted by a TCT codon encoding serine in chromosome II. Tyrosine and serine are polar, non-charged hydrophilic amino acids, and therefore both duplicate copies of *cox2* gene are assumed to be functional. In addition, both of the *trnD* and *trnH* genes on chromosomes I and II are also presumed to be functional because the nucleotide substitution had no effect on the tRNA secondary structure (Supplementary Fig. S1). Low levels of sequence difference between homologous gene copies on chromosome I and II suggests that a bipartite mitochondrial genome has evolved in this species relatively recently, although more work is needed to confirm this. The *cox2* genes on the two chromosomes of *R. karukerae* are 99.9% similar, whereas between *R. karukerae* and its putative closest three sequenced relatives (*Rhigonema thysanophora* [Rhigonematomorpha], *Ascaridia columbae* and *Cucullanus robustus*^22^), *cox2* sequence similarity ranges from 63% to 69%.

### Protein-coding genes and codon usage

Among the 12 PCGs, six genes (*cox2*, *nad5*, *nad6*, *nad2*, *cox3* and *nad4*) use ATA as the start codon, four (*cox1*, *nad3*, *nad4l* and *nad1*) start with ATT, and two (*atp6* and *cob*) start with ATG (Table 1). As a termination codon, seven genes (*cox1*, *cox2*, *nad3*, *nad6*, *nad4l*, *nad1* and *nad4*) were inferred to use TAA, two genes (*atp6* and *cob*) to use TAG, and three genes (*nad5*, *nad2* and *cox3*) to use the incomplete termination codon ‘T’.

The PCGs were biased towards T-rich codons (more than 2 Ts per triplet), similar to other chromadorean nematodes^16,19,21–23^. The three most commonly used codons from each chromosome were all T-rich: TTT (11.3%), TTA (8.4%) and ATT (6.2%) for chromosome I and TTT (11.3%), TTA (9.9%) and ATT (7.2%) for chromosome II (Supplementary Table S1). In contrast, the frequency of C-rich codons (two or more Cs per triplet) was only 3.3% of the total PCGs from chromosome I and 3.5% of the total PCGs from chromosome II.

### Transfer RNA gene and the non-coding region

Twenty-two tRNA gene sequences, ranging in size from 53 bp (*trnW*) to 70 bp (*trnV*), are inferred to fold into secondary structures of tRNAs (Supplementary Fig. S1, Table 1). Twenty of the tRNAs contain an amino-acyl stem of 7 bp and a DHU arm and anticodon stem, but lack a TΨC arm structure. In contrast, *trnS1* and *trnS2* have a TΨC arm and lack a DHU arm. These tRNA structures are commonly found in other nematode species^21^. The *trnD*, *trnG* and *trnH* genes were found on both chromosome I and chromosome II (Fig. 1, Table 1). The *trnD* and *trnH* sequences on chromosome I differed from their corresponding sequences (homologous genes) on chromosome II by a single nucleotide, but their putative secondary structure forms were identical (Supplementary Fig. S1).

On chromosome I, a non-coding region (designated NCR1) with a total length of 471 bp was found between *trnS1* and *nad4l*. On chromosome II, a non-coding region (designated NCR2) 489 bp in length was located between *trnV* and *trnP* (Fig. 1, Table 1). The A + T contents of the non-coding regions on chromosome I and chromosome II were 73% and 70.6%, respectively (Supplementary Table S2).

### Mitochondrial gene arrangement of *R. karukerae*

**Figure 2:**
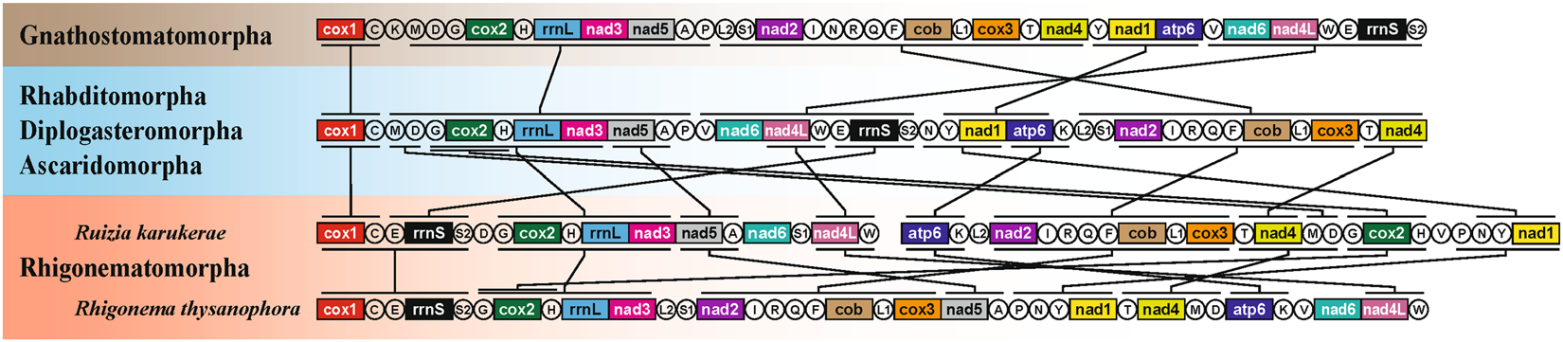
Linearized comparison of the mitochondrial gene arrangement of *Ruizia karukerae* and *Rhigonema thysanophora* (Rhigonematomorpha) and one species representative of Ascaridomorpha, Diplogasteromorpha, Gnathostomatomorpha and Rhabditomorpha, respectively. 22 tRNA genes are indicated by a single-letter abbreviation. The two leucine and two serine tRNA genes are marked according to their separate anticodon sequence, as L1 (*trnL1*-uag), L2 (*trnL2*-uaa), S1 (*trnS1*-ucu), and S2 (*trnS2*-uga). The non-coding regions are not shown.

The two species of Rhigonematomorpha for which mitochondrial genomes are now available (i.e., *R. karukerae* and *R. thysanophora*) share many gene clusters even though their gene order is not identical. Specifically, *nad4l*-*trnW*-*cox1*-*trnC*-*trnE*-*rrnS*-*trnS2*, *trnG*-*cox2*-*trnH*-*rrnL*-*nad3*, and *nad5*-*trnA* are shared between *R. thysanophora* and chromosome I of *R. karukerae*, and *atp6*-*trnK*, *nad2-trnI-trnR-trnQ-trnF-cob-trnL1-cox3*, *trnT-nad4-trnM-trnD*, *trnG-cox2-trnH*, and *trnP-trnN-trnY-nad1* are shared between *R. thysanophora* and chromosome II of *R. karukerae* (Fig. 2). Although mitochondrial phylogenies support a sister relationship among Rhigonematomorpha, Ascaridomorpha and Gnathostomatomorpha^22,24^, gene order in rhigonematomorphs is more similar to the most common gene order pattern among Ascaridomorpha, Diplogasteromorpha and Rhabditomorpha: they have many shared gene clusters: *cox1-trnC*, *trnM-trnD*, *trnG-cox2-trnH-rrnL-nad3*, *nad5-trnA*, *nad4l-trnW*, *trnE-rrnS-trnS2*, *trnN-trnY-nad1*, *atp6-trnK*, *nad2-trnI-trnR-trnQ-trnF-cob-trnL1-cox3* and *trnT-nad4*.

## Discussion

As presented in this study, the mitochondrial genome of *R. karukerae* consists of two circular chromosomes. There are a few other metazoan species in which the mitochondrial genome is known to be divided into multiple chromosomes. In such multipartite genomes, the size and the number of chromosomes vary widely. For example, *Brachionus plicatilis* and *B. koreanus* (rotifers), *Liposcelis bostrychophila* (booklouse), *Rhabditophanes* sp. KR3021 (nematode) and *Globodera ellingtonae* (nematode) all have two circular, evenly sized mitochondrial chromosomes^12,13,25–27^; however, the insect *Scirtothrips dorsalis* (thrip) has two chromosomes of very different sizes (14.3 kb and 0.9 kb)^28^. In contrast, the potato cyst nematodes (*Globodera pallida* and *G. rostochiensis*) have a least six circles^10,11,29^, and several lice species have mitochondrial genomes with 9 to 20 circles ranging from 1.5 to 5 kb^30–37^. In a few animals even the circular structure of the mitochondrial genome is not conserved: linearized mitochondrial genomes have been reported from an isopod (*Armadillidium vulgare*) and some cnidarians^38,39^.

The number of mitochondrial chromosomes in *R. karukerae* is the same as *G. ellingtonae* and *Rhabditophanes* sp.^12,13^, but the size of its two chromosomes (less than 8 kb each) is more similar to those of *G. pallida*, a nematode with at least six mitochondrial chromosomes (each 6.4–9.5 kb in size) or *Rhabditophanes* sp. (9.3 kb and 9.2 kb) rather than those of *G. ellingtonae* (17.8 kb and 14.4 kb)^10,12,13,29^. A common feature of *G. ellingtonae, Rhabditophanes* sp. and *G. pallida* is that they have abnormally long NCSs (non-coding sequences). The two mitochondrial chromosomes of *G. ellingtonae* have lengthy, intergenic NCSs between almost every gene (7 bp-8.1 kb for chromosome I and 1 bp-7.2 kb chromosome II)^13^. The longest NCSs in both chromosomes of *Rhabditophanes* sp. are each 2.6 kb^12^. In *G. pallida*, all six of its mitochondrial chromosomes contain many NCSs (3 to 7.4 kb in length)^10,29^. Abnormally lengthy NCSs in multipartite mitochondrial genomes are also found in some other metazoan groups: for example, two mitochondrial chromosomes of *B. plicatilis* (rotifer) have long NCSs (4.9 kb each)^25^ and *L. bostrychophila* (booklouse) has a relatively large number of intergenic NCSs (1–485 bp)^26^. In contrast, the NCSs of *R. karukerae* are more or less similar in size to those found in typical nematode species with a single mitochondrial chromosome (i.e., genes are abutting or the intergenic spaces are relatively short) (Fig. 1, Table 1).

Among nematodes with multipartite mitogenomes, there is considerable variation both in the characteristics of duplicated genes and the sequence divergence between duplicated copies. The duplicated genes found on both circular chromosomes (chromosome I and II) of *R. karukerae* comprise *cox2* and three tRNA genes (*trnD*, *trnG* and *trnH*), accounting for 856 bp (Fig. 1, Table 1), and their sequences are identical or nearly so: the two copies of *trnG* are the same, whereas the other genes (*cox2*, *trnD* and *trnH*) are nearly identical to their homologous copies, each differing by only a single base pair. In comparison, the duplicated region (2.6 kb) in each of the mitochondrial chromosomes of *Rhabditophanes* sp. is identical^12^, while in *G. ellingtonae* the two mitochondrial chromosomes share both an NCS and several copies of nonfunctional pseudogenes (highly degraded or truncated), with no homologous, functional genes shared between the two circles^13^. In *G. pallida*, several protein-coding genes are found on more than one circle, but many of the duplicated genes are nonfunctional due to frame-shift or nonsense mutations^29^. Features of the duplicated regions of mitochondrial chromosomes in other non-nematode groups are somewhat similar: the rotifer *B. plicatilis* has a duplicated region 4.9 kb in size containing the tRNA(L) genes and a non-coding region^25^, while in the booklouse *L. bostrychophila*, each 0.9 kb duplicated region is identical and includes three tRNA genes (*trnA*, *trnE* and *trnM*) and a putative control region^26^.

To explain how multipartite mitogenomes arise, various models have been suggested, such as intramolecular homologous recombination^40^, or genome replication^13^ followed by gene deletion^41^; however, a broad consensus has not yet been reached. Multipartite mitochondrial genomes with circular or linear forms have evolved independently in disparate, unrelated taxa (insects, rotifers, and nematodes [circular form]; sponges, hydras, box jellyfish [linear form])^42^, but it is still unclear how multiple chromosomes can arise from a single circular chromosome.

The two main arguments for why multipartite mitochondrial genomes exist are genetic drift and/or an unknown evolutionary advantage to having multiple mitochondrial chromosomes^26,43,44^ (but see^45^). There is no evidence favoring one of these arguments over another for *R. karukerae*, but there is some evidence that natural selection may favor shorter mitochondrial genomes. For example, in cultured human cells with both 11 kb and 16 kb mitochondrial circles, smaller circles became more numerous over time while the larger circles decreased in number^46^; and in heteroplasmic crickets, smaller mitochondrial genomes are transmitted to offspring more frequently than larger genomes^43^. Alternatively, multiple mitochondrial chromosomes may exist because a specific replisome gene (the mitochondrial single-strand binding protein, *mtSSB* that aids in DNA replication) was previously lost or mutated. The abnormality in this gene would prevent a full-size mitochondrial chromosome from being replicated, but would still allow smaller chromosomes to exist. In lice, it has been argued that the absence of this gene is responsible for multipartite mitochondrial genomes^33^. Although it is unknown exactly how or why multipartite genomes arise, previous work has noted a correlation with blood-feeding^30^ or a parasitic life-style^47^. All nematode species thus far recorded as having multipartite mitochondrial genomes, including the present study, are parasitic species (*G. ellingtonae*, *G. pallida*, *G. rostochiensis* [plant parasitic]; *Rhabditophanes* sp. KR3021, *R. karukerae* [animal parasitic]). However, comparatively few free-living nematode mitogenomes have been sequenced (compared to parasitic forms), and there is no clear evidence that parasitic life styles are correlated with multipartite mitochondrial genomes, nor has any work conclusively demonstrated that multipartite mitogenomes would be an advantage for parasites. Much more research is needed to better elucidate the evolutionary mechanisms leading to unusual mitochondrial genome structures.

Mitochondrial DNA genes have a relatively long history of use in phylogenetics^2^ and phylogeography^48,49^. More recently, phylogenetic comparisons of invertebrates based on complete mitochondrial genomes have been used for assessing deep relationships, and also for comparing species sharing more recent common ancestry^6,50–54^. This range of resolution is possible because mitochondrial genomes are composed of genes with very different rates of substitution. Faster evolving genes track more recent evolutionary events, whereas more conserved genes (and protein sequences) are informative for some deeper divergences. Disadvantages of mitochondrial DNA include that its multiple genes are inherited as a single locus, and certain groups of organisms show substantial nucleotide bias across genes. For nematodes, mtDNA genomes represent one of the main loci that have been used to infer phylogenetic relationships spanning the phylum. NCBI contains complete mitochondrial genomes for 176 nematode species. In contrast, nuclear ribosomal genes are a much more extensively sampled locus, with thousands of nematode species sequenced for 18S (SSU) rDNA^55,56^. This difference in taxon sampling between SSU and mitogenomes precludes detailed comparisons of phylogenetic results, but the main phylogenetic framework resulting from analysis of these separate loci is concordant^21,57^ despite some notable specific differences^16,19^ that will need to be tested through sampling of additional loci from nuclear genomes.

## Materials and Methods

### Specimen sampling and molecular methods

Nematode specimens were obtained from *Anadenobolus monilicornis* (millipedes) collected from the John Pennecamp Coral Reef State Park, Key Largo, Florida, USA by R. Carreno. The specimens were identified based on morphological features and measurements^58^. Total genomic DNA was extracted using a commercial kit (Epicentre MasterPure DNA Purification Kit; Epicentre Co.) following the manufacturer’s protocol. Four partial DNA fragments from four different genes (*cox1, rrnS, rrnL* and *nad5*) were amplified by polymerase chain reaction (PCR) using a universal primer set (LCO1490/HCO2198^59^) for *cox1* and three nematode-specific primer sets (Nema_12S_F/Nema_12S_R for *rrnS*, Nema_16S_F_2/Nema_16S_R for *rrnL* and Nema_ND5_F/Nema_ND5_R for *nad5*), designed directly from conserved regions of nematode mitochondrial genes (Table 2). PCR amplifications were carried out using TaKaRa Ex Taq (Takara) in a total volume of 50 ul containing 2 ul template DNA, 10 pmol of each primer, 1.25 u of Ex Taq polymerase, 1X Ex Taq buffer and 0.2 mM dNTP mixture, with the following amplification conditions: one initial denaturing step at 95 °C for 1 min followed by 35 cycles of denaturation at 95 °C for 30 s, annealing at 47 °C for 30 s, extension at 72 °C for 1 min, and final elongation at 72 °C for 10 min. Four primer pairs (RP1/RP2, RP3/RP4, RP5/RP6 and RP7/RP8) were designed from the sequences of the partial fragments (*cox1*, *rrnS*, *rrnL* and *nad5*) (Table 2) and used to obtain four overlapping long PCR fragments ranging from 1.5 kb to 2.7 kb: *cox1*-*rrnS* (1.5 kb), *rrnS*-*rrnL* (1.8 kb), *rrnL*-*nad5* (1.7 kb) and *nad5*-*cox1* (2.7 kb). Long PCR reactions consisted of 2 ul template DNA, 10 pmol of each primer, 2.5 unit LA *Taq* polymerase (TaKaRa), 1X LA Taq buffer, 0.4 mM dNTP mixture, 2.5 mM MgCl_2_ and 29.5 ul distilled water with the following amplification conditions: one cycle of initial denaturing at 95 °C for 1 min followed by 40 cycles of denaturation at 95 °C for 30 s, annealing and extension at 55 °C to 65 °C for 3 min to 10 min, followed by a final extension at 68 °C for 10 min. The amplified PCR products were purified using a QIAquick Gel Extraction Kit (QIAGEN Co.) following standard protocols. The sequences of the PCR-amplified fragments were determined for both strands using Big Dye Terminator Cycle-Sequencing (Applied Biosystems) and a primer walking strategy. The sequence of a complete strand of mtDNA was assembled by checking the sequences of the overlapping regions of the long PCR fragments and partial fragments obtained from the four different genes (*cox1*, *rrnS*, *rrnL* and *nad5*). Initially only a 7,659 bp contig (chromosome I) was obtained, containing six PCGs, two rDNAs, and nine tRNAs (Fig. 1). To locate the other genes, three partial fragments of three protein-coding genes missing from chromosome I (*cob*, *nad1* and *nad4*) were amplified using three nematode specific primer sets (Chroma_ND1_F_4/Chroma_ND1_R_2 for *nad1*, Chroma_ND4_F_1/Chroma_ND4_R_3 for *nad4* and Chroma_Cob_F_2/Chroma_Cob_R_2 for *cob*) (Table 2), and then sequenced. Using three species-specific primer sets (RP9/RP10, RP11/RP12 and RP13/RP14) designed from *cob*, *nad1* and *nad4* partial sequences (Table 2), three overlapping fragments were amplified by long-PCR and sequenced. The sequence of the complete strand of the second contig (chromosome II) was assembled by checking the sequences of the overlapping regions of the three long PCR fragments and the partial fragments obtained from the *cob*, *nad1* and *nad4* genes (Fig. 1).

### Gene annotation

The 12 mitochondrial protein-coding genes and two ribosomal RNA genes of *R. karukerae* were identified using the annotation program DOGMA^60^ and ORF finder (NCBI), and were confirmed by comparing nucleotide sequences with those from closely related nematodes. Putative secondary structures of 22 tRNA genes were inferred using the program tRNAscan-SE^61^ and verified by examining potential tRNA secondary structures and anticodon sequences.

### Data Availability

The datasets generated during and/or analysed during the current study are available from the corresponding author on reasonable request.

## Electronic supplementary material

Supplementary information
