# Supplementary material for: The bipartite mitochondrial genome of Ruizia karukerae (Rhigonematomorpha, Nematoda)
Source: Sci Rep. 2018 May 10;8:7482. doi: 10.1038/s41598-018-25759-0 (PMC5945635; doi:10.1038/s41598-018-25759-0)
Supplement: Supplementary file 1 — Supplementary information [file 41598_2018_25759_MOESM1_ESM.pdf]

Supplementary information

The bipartite mitochondrial genome of *Ruizia karukerae* (Rhigonematomorpha, Nematoda)

Taeho Kim[1], Elizabeth Kern[2], Chungoo Park[3], Steven A. Nadler[4], Yeon Jae Bae[1] & Joong-Ki Park[2]

[1]Division of Environmental Science and Ecological Engineering, College of Life Sciences and Biotechnology, Korea University, Seoul 02841, Republic of Korea

[2]Division of EcoScience, Ewha Womans University, 52 Ewhayeodae-gil, Seodamun-gu, Seoul 03760, Republic of Korea

[3]School of Biological Sciences and Technology, Chonnam National University, Gwangju 61186, Republic of Korea

[4]Department of Entomology and Nematology, University of California, Davis, CA 95616, USA

*Corresponding author

Joong-Ki Park

Address: Division of EcoScience, Ewha Womans University, 52 Ewhayeodae-gil, Seodamun-gu, Seoul 03760, Republic of Korea.

E-mail: jkpark@ewha.ac.kr

Supplementary Table S1. Genetic code and codon usage for the mitochondrial protein coding genes of *Ruizia karukerae*.

Codon	AA	No.	%	Codon	AA	No.	%	Codon	AA	No.	%	Codon	AA	No.	%
			**Chromosome I**								**Chromosome II**				
TTT	Phe	183	11.28	TAT	Tyr	75	4.62	TTT	Phe	229	11.30	TAT	Tyr	104	5.13
TTC	Phe	18	1.11	TAC	Tyr	22	1.36	TTC	Phe	35	1.73	TAC	Tyr	23	1.13
TTA	Leu	137	8.44	TAA	*	5	0.31	TTA	Leu	201	9.92	TAA	*	3	0.15
TTG	Leu	51	3.14	TAG	*	0	0.00	TTG	Leu	60	2.96	TAG	*	2	0.10
CTT	Leu	18	1.11	CAT	His	24	1.48	CTT	Leu	17	0.84	CAT	His	30	1.48
CTC	Leu	5	0.31	CAC	His	2	0.12	CTC	Leu	3	0.15	CAC	His	1	0.05
CTA	Leu	22	1.36	CAA	Gln	9	0.55	CTA	Leu	20	0.99	CAA	Gln	10	0.49
CTG	Leu	7	0.43	CAG	Gln	7	0.43	CTG	Leu	9	0.44	CAG	Gln	21	1.04
ATT	Ile	100	6.16	AAT	Asn	58	3.57	ATT	Ile	146	7.20	AAT	Asn	60	2.96
ATC	Ile	18	1.11	AAC	Asn	5	0.31	ATC	Ile	22	1.09	AAC	Asn	10	0.49
ATA	Met	78	4.81	AAA	Lys	28	1.73	ATA	Met	96	4.74	AAA	Lys	41	2.02
ATG	Met	32	1.97	AAG	Lys	15	0.92	ATG	Met	24	1.18	AAG	Lys	11	0.54
GTT	Val	55	3.39	GAT	Asp	28	1.73	GTT	Val	74	3.65	GAT	Asp	20	0.99
GTC	Val	7	0.43	GAC	Asp	8	0.49	GTC	Val	8	0.39	GAC	Asp	5	0.25
GTA	Val	46	2.83	GAA	Glu	20	1.23	GTA	Val	58	2.86	GAA	Glu	29	1.43
GTG	Val	24	1.48	GAG	Glu	11	0.68	GTG	Val	35	1.73	GAG	Glu	30	1.48
TCT	Ser	58	3.57	TGT	Cys	17	1.05	TCT	Ser	77	3.80	TGT	Cys	18	0.89
TCC	Ser	6	0.37	TGC	Cys	0	0.00	TCC	Ser	5	0.25	TGC	Cys	0	0.00
TCA	Ser	5	0.31	TGA	Trp	25	1.54	TCA	Ser	9	0.44	TGA	Trp	26	1.28
TCG	Ser	6	0.37	TGG	Trp	15	0.92	TCG	Ser	6	0.30	TGG	Trp	23	1.13
CCT	Pro	25	1.54	CGT	Arg	11	0.68	CCT	Pro	36	1.78	CGT	Arg	19	0.94
CCC	Pro	1	0.06	CGC	Arg	0	0.00	CCC	Pro	2	0.10	CGC	Arg	0	0.00
CCA	Pro	6	0.37	CGA	Arg	0	0.00	CCA	Pro	7	0.35	CGA	Arg	1	0.05
CCG	Pro	1	0.06	CGG	Arg	3	0.18	CCG	Pro	3	0.15	CGG	Arg	2	0.10
ACT	Thr	38	2.34	AGT	Ser	45	2.77	ACT	Thr	48	2.37	AGT	Ser	49	2.42
ACC	Thr	3	0.18	AGC	Ser	13	0.80	ACC	Thr	4	0.20	AGC	Ser	18	0.89
ACA	Thr	10	0.62	AGA	Ser	39	2.40	ACA	Thr	10	0.49	AGA	Ser	43	2.12
ACG	Thr	1	0.06	AGG	Ser	18	1.11	ACG	Thr	5	0.25	AGG	Ser	22	1.09
GCT	Ala	27	1.66	GGT	Gly	57	3.51	GCT	Ala	26	1.28	GGT	Gly	45	2.22
GCC	Ala	5	0.31	GGC	Gly	3	0.18	GCC	Ala	10	0.49	GGC	Gly	8	0.39
GCA	Ala	6	0.37	GGA	Gly	31	1.91	GCA	Ala	7	0.35	GGA	Gly	23	1.13
GCG	Ala	7	0.43	GGG	Gly	23	1.42	GCG	Ala	5	0.25	GGG	Gly	33	1.63

* Termination codon

Supplementary Table S2. Nucleotide composition of the mitochondrial genome of *Ruizia karukerae*.

Nucleotide sequence	Length (bp)	A (%)	C (%)	G (%)	T (%)	A+T (%)	G+C (%)	Length (bp)	A (%)	C (%)	G (%)	T (%)	A+T (%)	G+C (%)
	**Chromosome I**							**Chromosome II**						
Entire sequence	7,659	29.0	9.5	16.8	44.7	73.7	26.3	7,647	27.5	9.8	17.2	45.5	73.0	27.0
Protein coding sequence	4,854	26.3	9.5	18.1	46.1	72.4	27.6	6,066	26.1	9.8	17.1	26.9	73.1	26.9
Codon position*														
1st	1,618	31.0	8.7	22.1	38.2	69.2	30.8	2,022	30.1	9.0	20.6	40.4	70.5	29.5
2nd	1,618	19.3	12.7	18.5	49.5	68.8	31.2	2,022	19.5	12.9	16.3	51.3	70.8	29.2
3rd	1,618	28.6	7.2	13.7	50.6	79.1	20.9	2,022	28.7	7.6	14.3	49.4	78.1	21.9
Ribosomal RNA gene	1,631	35.4	8.3	14.1	42.1	77.6	22.4	-	-	-	-	-	-	-
Transfer RNA gene	506	33.4	11.1	15.2	40.3	73.7	26.3	910	34.0	8.5	18.2	39.3	73.3	26.7
Non-coding region	471	27.0	11.5	15.5	46.1	73.0	27.0	489	30.3	11.7	17.8	40.3	70.6	29.4

*Termination codons were not included

Chromosome I

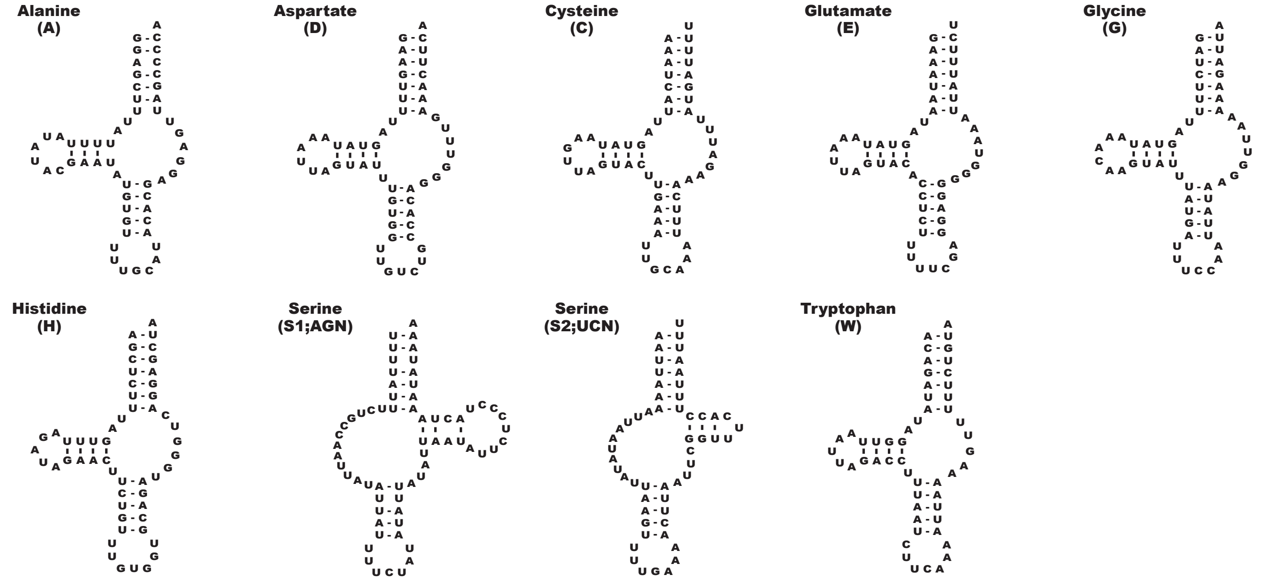

Chromosome II

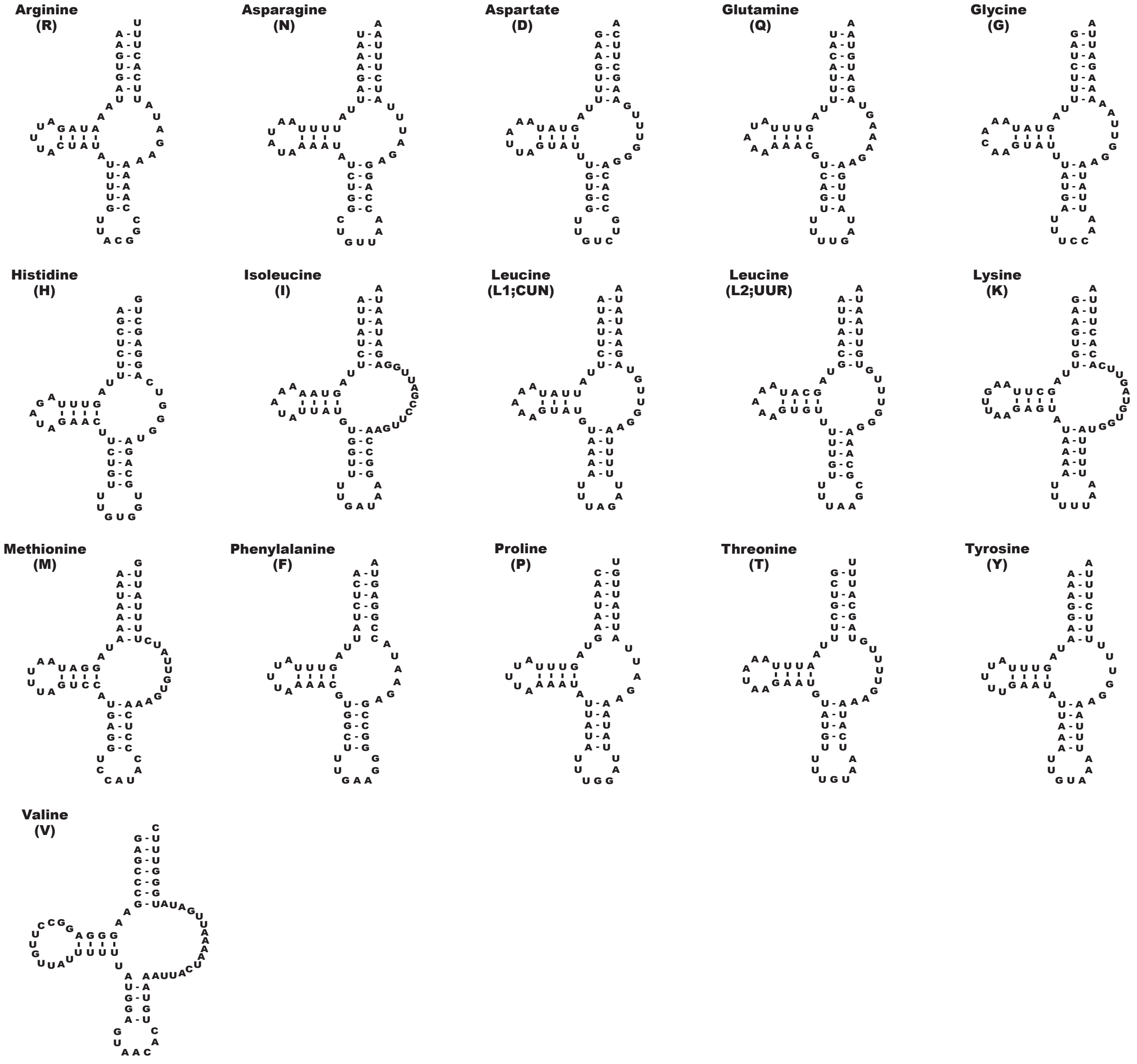

Supplementary Figure S1. Putative secondary structures of the 25 mitochondrial tRNAs of *Ruizia karukerae*. *trnD*, *trnG* and *trnH* are present on both chromosomes.
